# In-Vivo Imaging of the Drosophila Wing Imaginal Disc over Time: Novel Insights on Growth and Boundary Formation

**DOI:** 10.1371/journal.pone.0047594

**Published:** 2012-10-16

**Authors:** Ulrike Nienhaus, Tinri Aegerter-Wilmsen, Christof M. Aegerter

**Affiliations:** 1 Physik-Institut, University of Zurich, Zurich, Switzerland; 2 Institute of Molecular Life Sciences, University of Zurich, Zurich, Switzerland; University of Dayton, United States of America

## Abstract

In developmental biology, the sequence of gene induction and pattern formation is best studied over time as an organism develops. However, in the model system of Drosophila larvae this oftentimes proves difficult due to limitations in imaging capabilities. Using the larval wing imaginal disc, we show that both overall growth, as well as the creation of patterns such as the distinction between the anterior(A) and posterior(P) compartments and the dorsal(D) and ventral(V) compartments can be studied directly by imaging the wing disc as it develops inside a larva. Imaged larvae develop normally, as can be seen by the overall growth curve of the wing disc. Yet, the fact that we can follow the development of individual discs through time provides the opportunity to simultaneously assess individual variability. We for instance find that growth rates can vary greatly over time. In addition, we observe that mechanical forces act on the wing disc within the larva at times when there is an increase in growth rates. Moreover, we observe that A/P boundary formation follows the established sequence and a smooth boundary is present from the first larval instar on. The division of the wing disc into a dorsal and a ventral compartment, on the other hand, develops quite differently. Contrary to expectation, the specification of the dorsal compartment starts with only one or two cells in the second larval instar and a smooth boundary is not formed until the third larval instar.

## Introduction

In the recent past, the focus of many biological investigations has shifted from a single gene perspective to one concerned with a mathematical modeling of the process in question [1]. In such a Systems Biology approach, the main goal is to be able to model a biological process at many levels. One of the key features of such models is that they describe the entire process quantitatively and therefore cannot rely solely on data from completed gene expression patterns [2]. Rather, to accurately model a developmental process by predicting the sequence of events, temporal information on gene induction and patterning, as well as growth, is requisite. This implies that temporal data is much more suitable for testing many of the quantitative models now available for describing growth and patterning.

One of the most prominent model systems for studying both patterning and growth is the wing imaginal disc of Drosophila [3]. This is a simple two-layered epithelial tissue which forms the adult wing’s precursor organ in the larval stage of development, turning into the adult wing during morphogenesis [4]. Several interesting problems in patterning and growth control in the wing disc exist which have so far mostly been studied on the basis of gene regulation at the end of larval development. However, it has become clear that a further understanding of these problems can only be attained by studying the temporal evolution of the processes. In growth control, for instance, models have implicated the influence of temporally varying growth factor expression as a way of explaining uniform growth and termination of growth [5]. This needs to be tested on time dependent data of the expression of said growth factors. Similarly, mechanical feedback models have been proposed for the explanation of uniform growth and growth termination [6]–[8], which predict a temporal development of growth and the buildup of mechanical forces. Again, these models need to be tested against time dependencies from the biological system. In other words, quantitative models on growth control predict time sequences which need to be studied experimentally.

In terms of patterning, the temporal development of morphogen expression patterns is of great importance. In the Drosophila wing, for instance, there is scale invariance in the vein pattern formed which has been associated with the scaling of morphogen gradients in the wing disc [9], [10]. A theoretical description of a mechanism for such a scaling expression pattern necessarily involves a modeling of the temporal evolution of the relevant morphogen gradients and the corresponding gene interaction network [11]. Again, such models can only be tested properly when temporal information on the expression patterns is available.

There are three possible ways of obtaining the necessary temporal data of a developing wing disc. The first possibility is to grow the tissue in vitro using cell culturing methods. Though there is progress in these techniques [12], [13], the available time window is still far below the full developmental cycle. Moreover, such methods may be difficult to realize until the role that external factors play has been assessed in greater detail. External factors such as possible mechanical forces may become especially challenging to in vitro culture methods, since dissection necessarily removes all external forces.

A second potential method is the dissection of discs at various time points and the subsequent study of e.g. the gene expression patterns at these different time points [9], [14]. This has recently been implemented in a few cases, however the variation between larvae may affect the results of such studies. In addition, growth effects and possible external growth factors cannot be assessed in this way.

**Figure 1:**
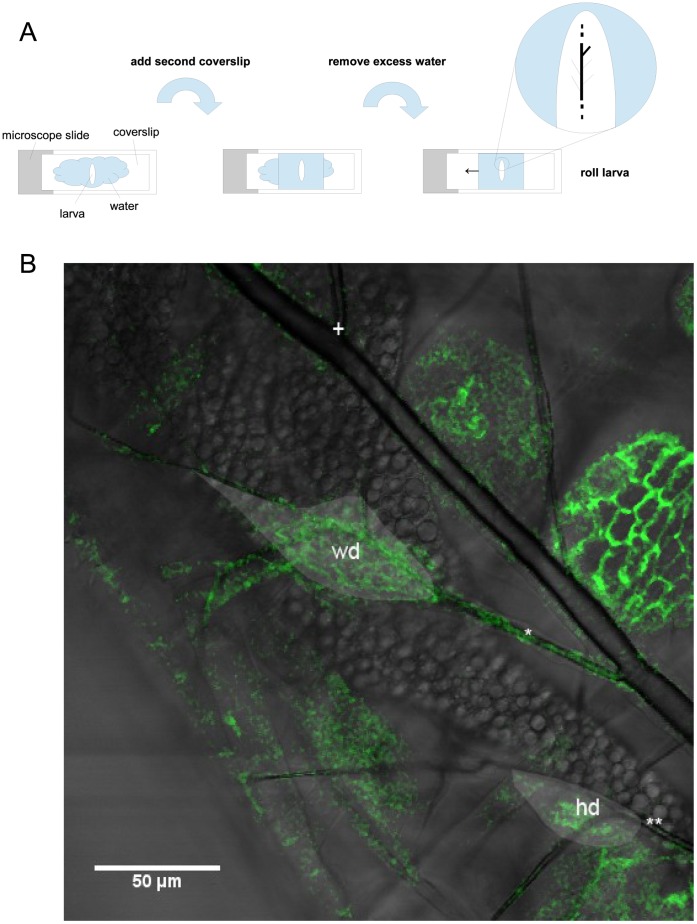
Illustration of the method of locating the wing and haltere discs. A: Schematic of the process for imaging the larvae and locating the wing and haltere discs. B: A section of the left dorsal trunk, main tracheal branch of a larva showing the positions of the wing(wd) and haltere(hd) discs. The discs are indicated by the lighter areas, the cell outlines are fluorescently marked by GFP fused to E-Cadherin. The thick tracheal branch which connects the right and left main tracheal branches can be seen at the top (+). The wing disc is connected to the first left side branch (*), counting from (+). The haltere disc is located on the second side branch (**).

The third possibility lies in the imaging of the developmental process within a live larva. In the past, temporal information on the development of imaginal discs of Drosophila from in-vivo imaging has not been available. This is in part due to the fact that imaging a tissue within a living larva has proven difficult. Besides being in constant motion, the larval tissues and the cuticle are turbid, hindering high resolution imaging.

In this paper, we present a technique for in-vivo imaging which allows us to gather information about the expression patterns of different genes, as well as the overall growth of imaginal tissue, throughout the entire larval stages of Drosophila. Applying our method of in-vivo imaging to the wing and haltere imaginal discs of Drosophila, we explore two different aspects of development. To study growth, we assess apical disc area over time, finding great variability between larvae. Unexpectedly, we also find that there are mechanical forces stretching the wing disc within the larva. To study patterning, we investigate the expression patterns of the genes engrailed (en) and apterous (ap) over time. The expression of these genes is known to specify different compartments in the wing disc, where engrailed is only expressed in the posterior compartment of the disc [15] and apterous is only expressed in the dorsal compartment of the disc [16]. The expression pattern and sequence of engrailed is well established from the embryonic through the larval stages [17]. Similarly, the control of a smooth boundary is established on a molecular level and has recently been modeled theoretically as well to indicate a component of mechanical tension leading to increased stability [18]. Our data are able to reproduce these known properties of en expression within the time evolution of single larvae. In the case of the D/V boundary, much is known about the control of the formation of a smooth boundary [19]–[21] and the corresponding molecular mechanisms. However, the induction of the expression of apterous [22]–[24] and hence the initial origin of the D/V boundary is far less well understood. Our data shed new light on the establishment of the D/V boundary, which we for instance do not find to be established as a smooth boundary until the third instar.

**Figure 2:**
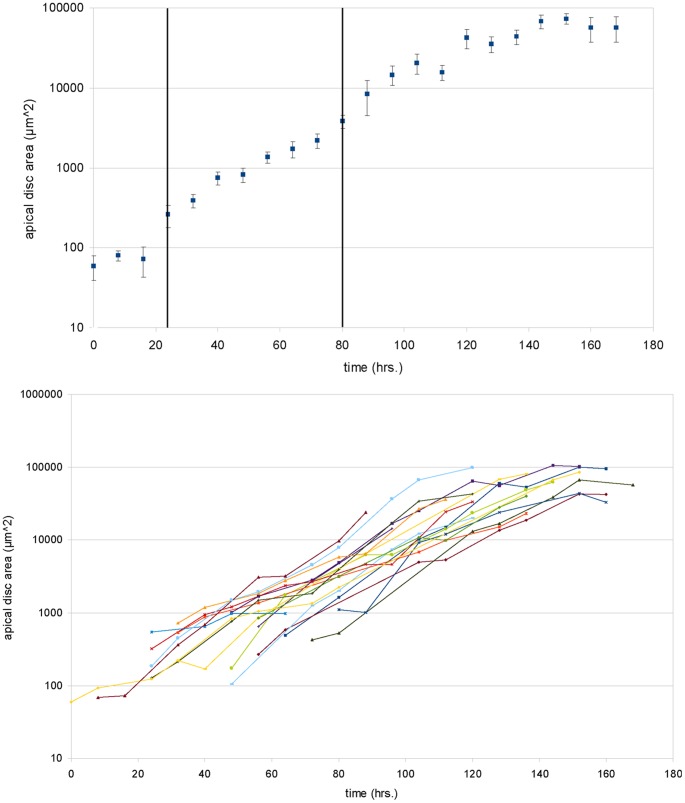
Growth curves of wing disc area during the larval stages. A: Semi-logarithmic plot of the average apical disc area as a function of time. The times were normalized, such that the first point in the third instar coincided with 80 hrs. The first instar with moderate growth ends after about 24 hrs. In the second (24–72 hrs.) and early third instars, growth is nearly exponential. In the late third instar the slope of the line decreases, indicating a decrease in the average growth rate. The error bars indicate the standard error of the respective time point. Note that in these experiments the larvae are kept at 22(1) °C, which explains the longer time periods of the different growth phases. The vertical lines delineate different instar stages of the larvae. B: Semi-logarithmic plot of the apical disc area for different larvae over time. The average of these growth curves yields the graph shown in part A. Each larva develops individually, yet there is generally moderate growth in the first instar, exponential growth in the second and early third and a decrease in the growth rate in the late third instar. The differences in the growth rates of individual larvae indicate that external factors play a role in wing disc development.

## Results

### 2.1. Sample Preparation and Imaging Protocol

In-vivo imaging in Drosphila larvae has been attempted before but has so far been used primarily to assess synapse development in structures that are closer to the cuticle than the imaginal discs [25]–[27]. Thus, so far no results for the growth and patterning of an organ through the entire time of development exist. Here, we present a method with which the taking of such data as well as data on the properties of growth and boundary formation becomes possible. Making use of the fact that the cuticle of Drosophila larvae becomes almost transparent in water due to a matching of the refractive index, as well as an immobilization technique, we have devised a minimally invasive method to be able to observe snapshots of these processes throughout the entire larval stage. Results on the wing and the haltere disc will be presented below.

**Figure 3:**
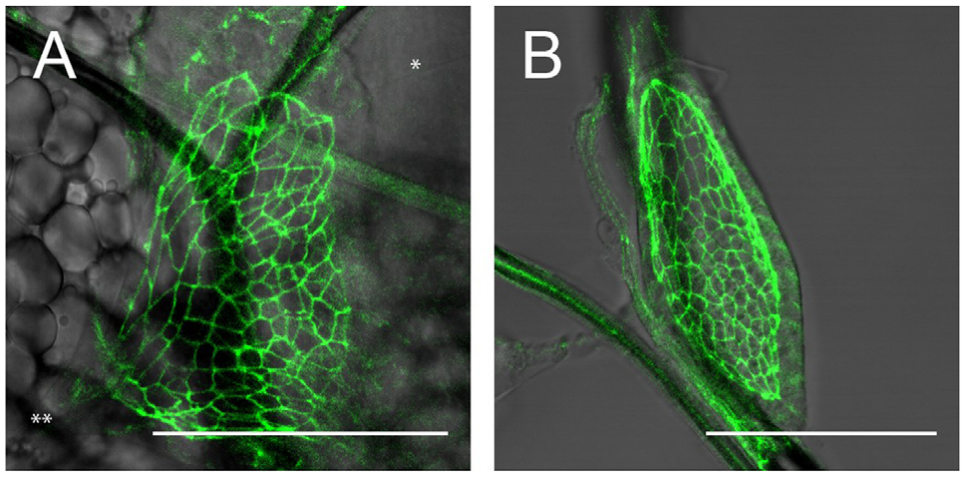
Images of a wing disc, where the apical cell outlines of the disc proper are marked by GFP fused to E-Cadherin. **A:** wing disc in vivo at the beginning of the third instar. In this image, several sources of mechanical force can be discerned. A tiny thread is attached to the wing disc on the posterior side (*). Also, the large muscle fiber between the wing, leg and haltere discs is clearly exerting a substantial force on the disc at (**). The cell outlines are correspondingly distorted. **B:** the same wing disc after dissection. Due to the dissection, the external force from the muscle fiber and the thread has been removed and the shape of the disc as well as that of the cell outlines has relaxed, showing a marked difference to A. The scale bar is 50 µm.

The appeal of the technique for in-vivo imaging we introduce here lies in its simplicity. No special equipment is needed and larvae are ready for imaging within a very short time, providing an alternative to previous methods using anesthetics such as desflurane or ether [25]–[27].

**Figure 4:**
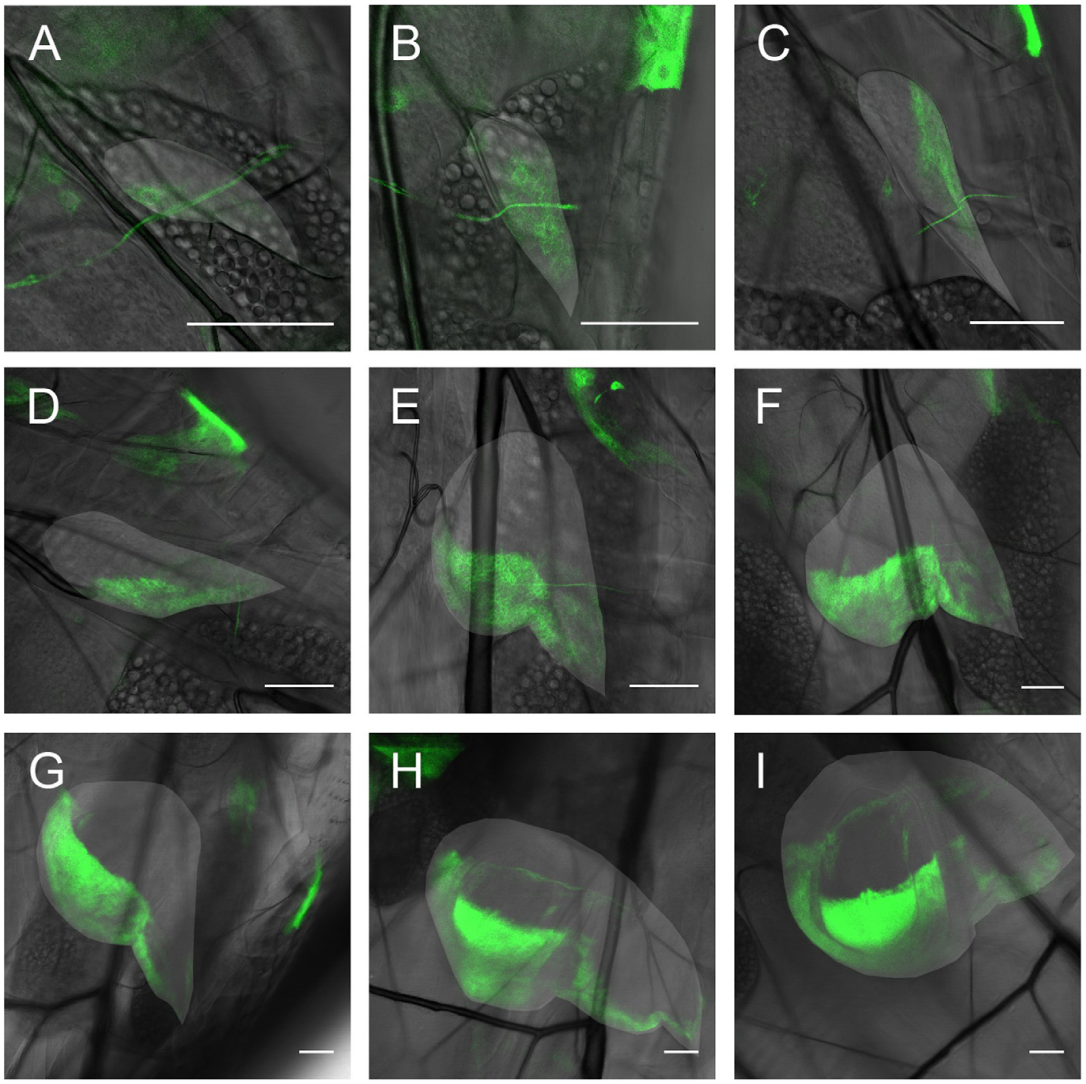
Engrailed expression pattern in the cells of a left wing disc over time, in flies expressing UAS CD8-GFP under the control of enGAL4. The wing disc is indicated by the lighter area in each image. **A:** first instar wing disc already showing an engrailed expression pattern, indicating the posterior compartment. The A/P boundary is smooth. **B:** same wing disc the next day (about 16(1) h after the first image (AFI)). The larva molted and is in the second instar. **C:** 24(1) h. AFI. In this image the disc is in a different position, hence the atypical orientation of the posterior compartment. **D:** 40(1) h. AFI. **E:** 48(1) h. AFI. After molting again, the larva is now in the third instar. The typical wing disc shape and engrailed pattern can now be recognized. **F:** 64(1) h. AFI. **G:** 72(1) h. AFI. **H:** 88(1) h. AFI. **I:** 96(1) h AFI. Note the kink in the expression pattern seen in subfigures H and I, as well as the additional expression of en in a line in the A compartment of the disc. The scale bar is 50 µm in all images.

In practice, to prepare the larvae for imaging they were first placed in a drop of water on a coverslip on top of a microscope slide. A second coverslip (thickness 170 µm) of a size appropriate to the larva’s size was then placed on top of the larva (see Fig. 1A). The size of the second coverslip limits the maximum capillary pressure possible for immobilization of the larvae. Due to the increase in mobility of a larva with size, the coverslip has to be adjusted to larval size. Typically 18×18 mm^2^ coverslips were used for first and second instar larvae, larger coverslips for third instar larvae. Excess water was carefully removed from between the coverslips using a paper tissue until the larva was immobilized by the capillary pressure of the remaining water. Thus combining the amount of water and the size of the cover slip gives a well adjustable force for the immobilization of the larvae. Due to the fact that the refractive indices of the coverslip and the water, as opposed to the refractive index of air, better match the refractive index of the larval cuticle, the cuticle loses its turbid quality, becoming transparent.

**Figure 5:**
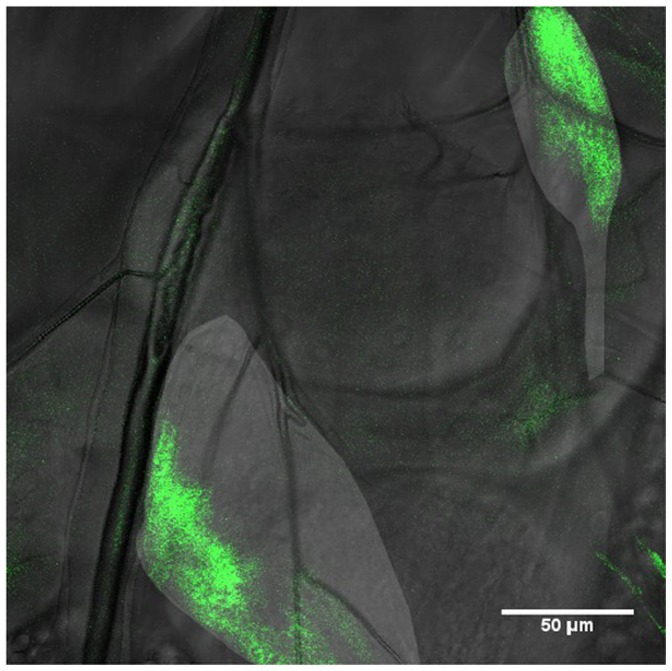
Image of the wing and haltere discs in flies expressing UAS CD8-GFP under the control of enGAL4. This shows the respective engrailed expression patterns. The lighter areas indicate the imaginal disc outlines. In the wing disc (lower left, located above the tracheal branch), the engrailed expression pattern, showing the posterior compartment, is oriented as expected in respect to the larva. In the haltere disc (upper right, located below the tracheal branch) the engrailed expressing cells are oriented quite differently in this stage, although they show the same orientation as the wing disc in later larval stages. This change in orientation over time may be induced by the muscle fiber between the wing, leg and haltere discs.

The larva’s position could now be corrected if necessary to give a clear view of e.g. the left main tracheal branch. This was done by carefully moving one coverslip against the other to roll the larva about its anterior-posterior axis until the desired tracheal branch was directly beneath the top coverslip. The immobilized larva was thus placed under an upright Leica SP1 confocal microscope. The imaginal disc of interest could be located by following the tracheal branches in view. The wing disc is located on the second thoracic lateral tracheal branch off of the main tracheal branch, the haltere disc on the third lateral tracheal branch (see Fig. 1B).

**Figure 6:**
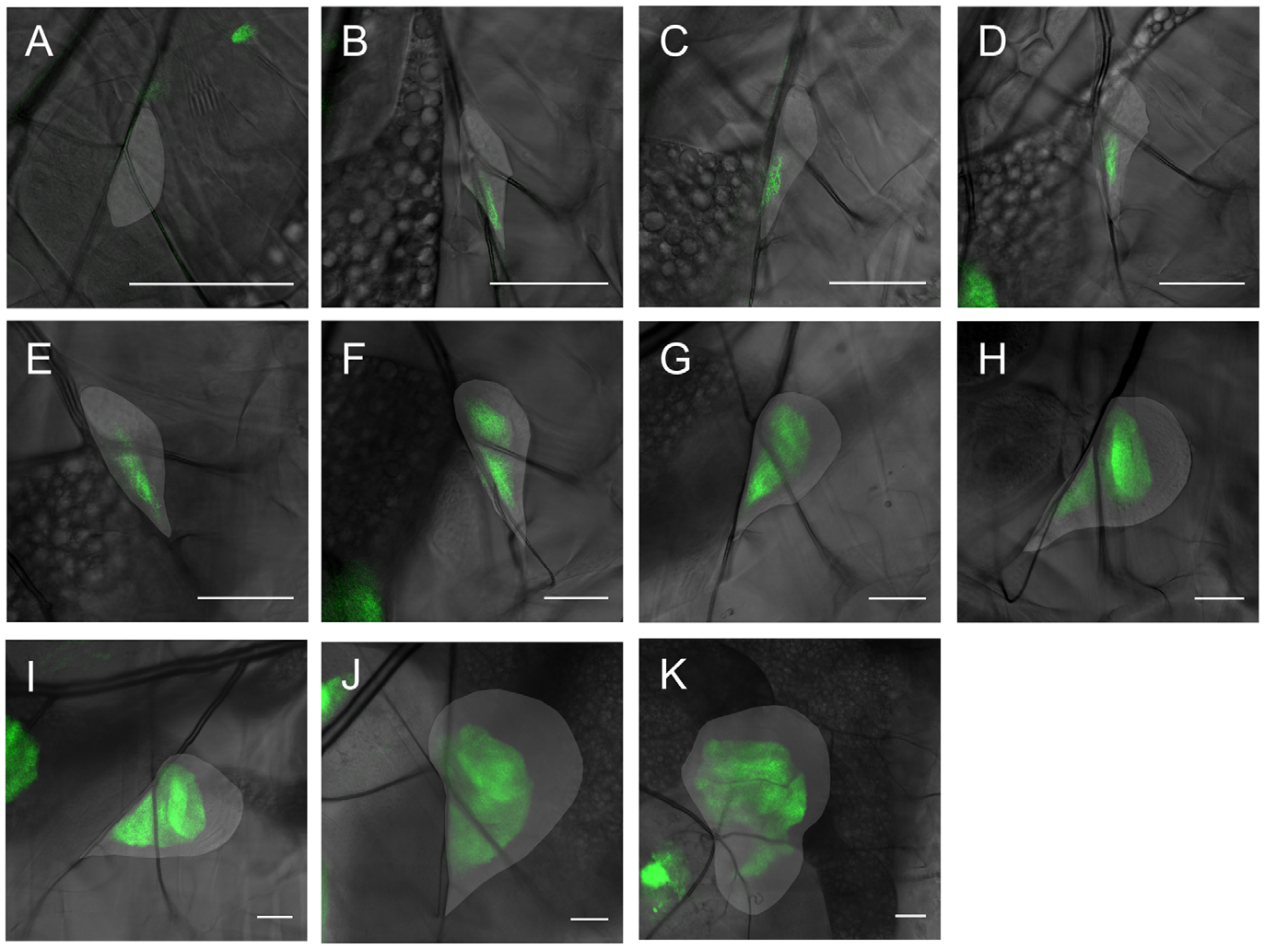
The apterous expression pattern in the left haltere disc over time in flies expressing UAS CD8-GFP under the control of apGAL4. The haltere disc is indicated by the lighter area. **A:** first instar haltere disc, without ap expression. **B:** same haltere disc the next day (about 16(1) h after the first image (AFI)). The larva has entered the second instar and now shows expression of apterous. **C:** 24(1) h AFI. **D:** 40(1) h AFI. **E:** 48(1) h AFI. **F:** 64(1) h AFI. The larva is in the third instar. **G:** 72(1) h AFI. In this image it can be seen that the D/V boundary is not yet smooth. **H:** 88(1) h AFI. The D/V boundary is now smooth and well-defined. **I:** 96(1) h AFI. **J:** 112(1) h AFI. **K:** 120(1) h AFI. The scale bar is 50 µm in all images.

A further reduction in turbidity of the obtained images is achieved by the use of a confocal microscope, both in fluorescence and transmission mode to also assess the physiology of the imaginal discs. Transmission and fluorescent images were taken at reduced laser power (Ar ion laser at 30% power setting corresponding to roughly 0.1 mW) as a precaution to prevent phototoxicity, though the light intensity did not appear to affect the well-being of the larva in any way. Also, imaging times were limited to a maximum of 5 to 10 minutes to ensure that the larva was exposed to as little stress as possible. Survival rates were impaired if imaging times exceeded 10 to 15 minutes.

**Figure 7:**
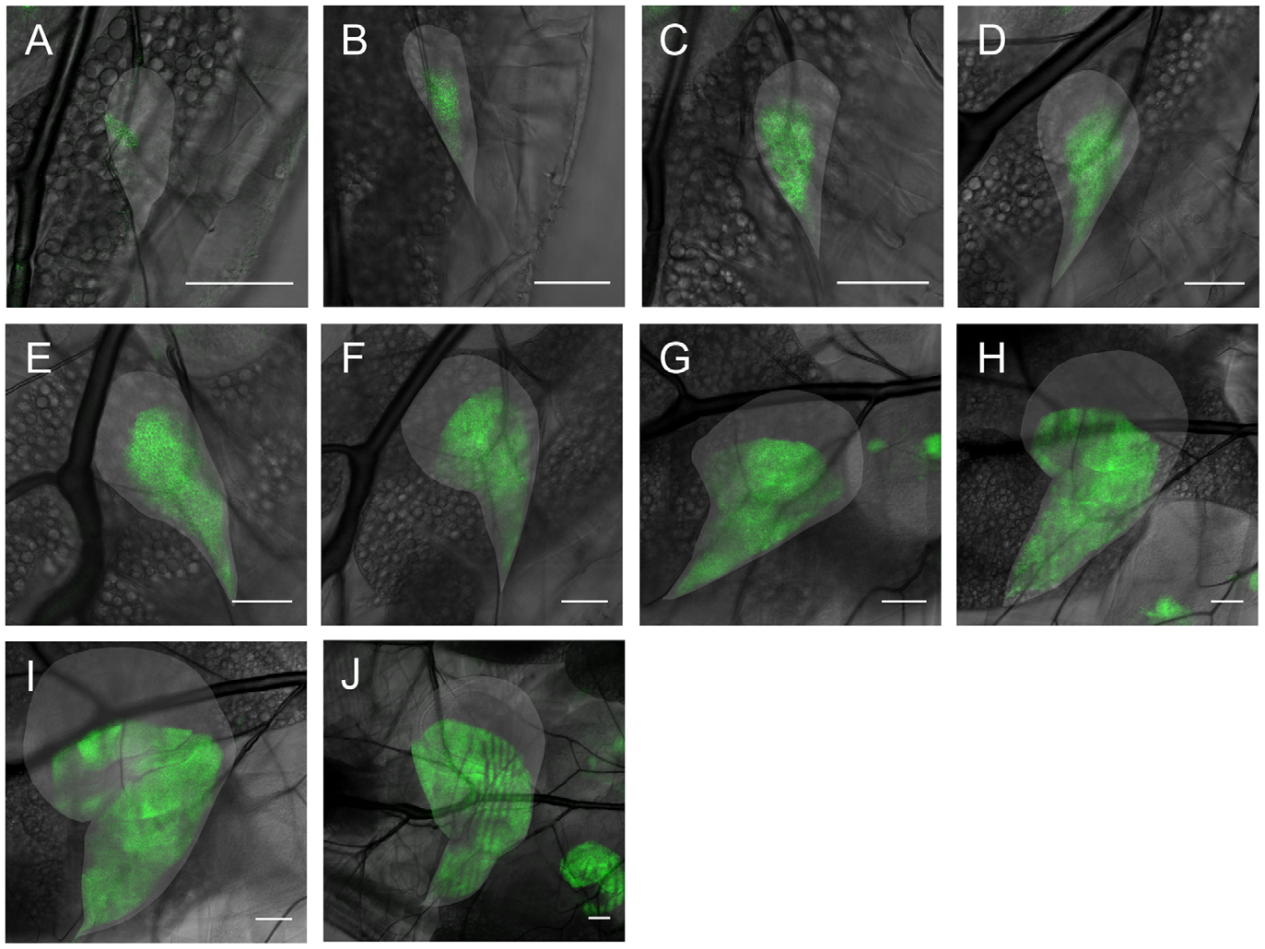
The apterous expression pattern in the left wing disc over time in flies expressing UAS CD8-GFP under the control of apGAL4. The wing disc is indicated by the lighter area. **A:** second instar wing disc, with first ap expression. **B:** same wing disc the next day (about 16(1) h after the first image (AFI)). **C:** 24(1) h AFI. **D:** 40(1) h AFI. The larva is in the third instar. In this and the following image it can be seen that the D/V boundary is not yet smooth but rather appears to be determined by non-oriented cell divisions**. E:** 48(1) h AFI. **F:** 64(1) h AFI. The D/V boundary is now smooth and well-defined. **G:** 72(1) h AFI. **H:** 88(1) h AFI. **I:** 96(1) h AFI. **J:** 112(1) h AFI. The scale bar is 50 µm in all images.

After imaging, the larva was released by adding water between the coverslips, thus gradually decreasing the capillary pressure. If normal feeding activity was resumed immediately following release, survival rates of the larvae were high (>80%).

**Figure 8:**
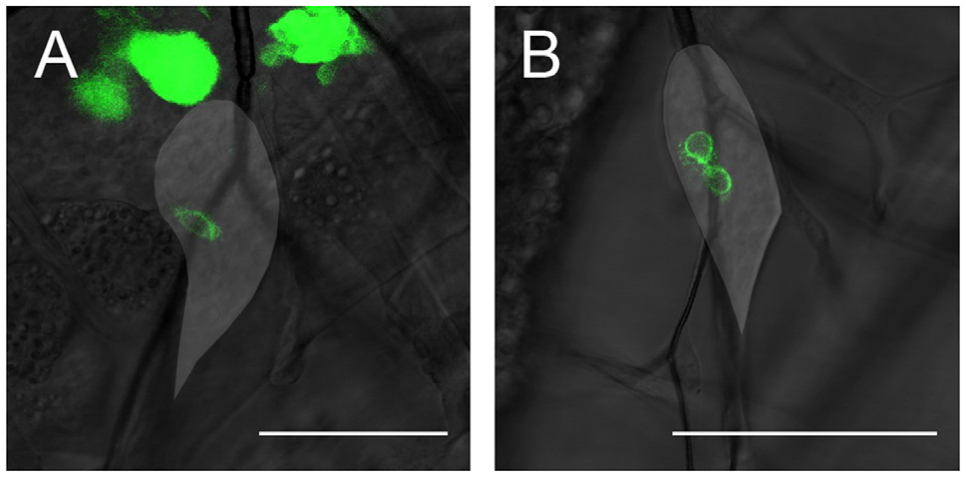
Two in vivo images showing the first apterous-expressing cells in the wing discs of two flies expressing UAS CD8-GFP under the control of apGAL4. The wing disc outlines are indicated by the lighter areas. **A:** In this wing disc, only a single cell shows apterous expression, indicating that although apterous is a marker for the dorsal compartment, the D/V boundary is not established simultaneously with the first apterous expression. **B:** two cells show apterous-expression. The rather typical position of the first apterous-expressing cells near the tracheal fork is not in accordance with a direct control of apterous via wingless and vein. The scale bar is 50 µm.

Larvae were imaged twice a day with a time span of about 8 hours between images, however more frequent imaging is possible and we also imaged wing disc development every 4 hours in some cases. In between rounds of imaging, the larvae were kept singly at room temperature on standard fly medium meaning that individual development could be followed.

### 2.2. Growth Properties of the Wing Imaginal Disc

To assess whether larvae developed normally, we studied the area of the wing disc over time. This was done using flies where the apical side of the cells was fluorescently marked using GFP fused to E-Cad. Such data has been obtained before by averaging disc sizes for wing discs dissected at specific time points from many individuals. When averaging the apical areas found in different individuals we find results similar to those of [14], thus showing that larvae develop normally while they are being imaged over time. This is shown in Fig. 2A, where the average apical disc area is shown on a semi-logarithmic plot as a function of time. The slope of this curve gives the average growth rate, which can be seen to decrease as the final size of the disc is reached. In addition, we observe that in the first instar phase the disc cells grow, but do not divide (data not shown), as is known from histological data [28]. During the late second and early third instar phase, the disc size grows roughly exponentially as is seen by the nearly straight line in the graph. Thus, from the data of the growth of the area of the wing disc throughout the whole larval stage, we conclude that the larvae develop normally during the entire imaging process.

Due to the fact that it is possible to follow single individuals over time using in-vivo imaging, we can plot the same data without averaging the sizes for each time point. This is shown in Fig. 2B, where the time dependence of the disc area is shown for single discs. These discs show a similar behavior over time, with some growth in area in the first instar, though without cell divisions, roughly exponential growth in the late second and early third instars and a termination of growth at the end of the third instar. However, the exact growth rates differ between larvae. Moreover, they vary over time in a single larva, indicating that each larva develops individually, yet in the same general way. Due to this variation in the development of different larvae, we have chosen to normalize the temporal axis onto a clear developmental point, namely the molt between the second and third instars. The instar stages can be determined from the physiology of the tracheal system in the larvae, which is also determined in the course of the imaging. Thus Fig. 2B shows that there is also variability in the duration of the different larval stages.

The individual development of wing discs within single larvae implies that the growth of imaginal discs is influenced by external factors, which is why we have chosen to study the overall physiology of wing imaginal discs at points of strong change in the individual growth rate. Such external influences may e.g. be mechanical forces, which have recently been implicated in the control of growth and growth termination [6]–[8]. Using in-vivo imaging of single larvae over time, it is possible to assess the presence of mechanical forces experimentally.

Since we are using a confocal microscope, as discussed above, it is possible to obtain transmission images simultaneously with fluorescence images. The transmission images allow the detection of non-fluorescent sources of external mechanical stresses acting upon the wing and haltere discs. Specifically for the wing disc, we have in this way discerned several sources of mechanical forces.

As was previously described in [29], muscle connections exist between the wing, leg and haltere discs. The muscle fiber which is attached to the wing disc in the anterior-ventral compartment appears to physically induce a considerable amount of stretching on the wing disc around the time of the second molt as can be seen in Fig. 3A. This mechanical stress can be observed not only in the overall shape of the wing disc, but also in the shapes of individual cells (cell shapes outlined by a ubiquitously expressed fusion protein of E-Cadherin with GFP), which change in reaction to this external stress. In order to demonstrate that the observed distinct change in cell shape is due to a pulling force, we dissected the wing disc previously imaged in vivo to remove external stress. The dissected wing disc is shown in Fig. 3B, where the cell shapes look relaxed. This verifies that the muscle fiber does indeed exert a pulling force on the wing disc at this time. Finally, the force is unlikely to be induced by the experimental protocol, as the strong stretching only and reproducibly appears in the second half of the second and the beginning of the third larval instars. These forces are acting n addition to growth induced stresses present at late third instar stages [30].

Besides this muscle fiber, marked by a double asterisk (**) in Fig. 3, forces are also exerted on the wing disc during other periods of the second larval instar by very small threads which appear to attach only to the posterior side of the wing disc, marked by a single asterisk (*) in Fig. 3. Moreover, the trachea as well as *twist*-Gal4 expressing cells near them seem to generate substantial forces during the first and early second instars.

Thus using the individual time resolved growth curves of wing discs, we find that there are strong increases in growth rates for instance around the molts, but also in the beginning of the third instar. At these times we have also found substantial external forces exerted on the wing disc in vivo, mainly by large muscle fibers, as discussed above.

### 2.3. Temporal Development of Boundary Formation

Having assessed that our method accurately describes wing disc growth, we were interested in whether wing disc patterning was also depicted correctly. As a check we chose to follow the pattern of the gene engrailed over time. Besides being well-established as a controlling factor in the development of the A/P boundary [17], this gene is known to be expressed in the wing disc from the beginning of the first larval instar on [15]. Also, the domain of engrailed expression leads to the formation of a smooth boundary via the induction of hedgehog [31], possibly in conjunction with an increase in the line tension between the boundary forming cells [18]. This means that it is well known what the domain of engrailed expression should look like during larval development. Hence, we can assess the applicability of the technique by studying the time dependence of the fluorescent activity of a GAL4-activated UAS-GFP fused to CD8, under the promoter of engrailed as a proxy of the engrailed domain.

Figs. 4 A–I show the fluorescent intensity of CD8-GFP as a measure of the expression of engrailed in the cells of the left wing disc for one larva from the first to the third larval instar. Images were taken twice a day with an interval of about 8 hours between the images. As expected, engrailed is already expressed in the posterior compartment in the first larval instar, comprising roughly half the cells of the wing disc. The A/P boundary is thus present from the beginning. Further inspection of Figs. 4 A–I shows that the boundary remains smooth throughout the entire larval stage. The pattern develops over time in the expected way, culminating in third instar wing discs which show the well-known A/P pattern. Thus, the genetic markers as well as the imaging method both do not seem to influence the development in a detrimental way.

A point of interest in connection with the gene engrailed appears with respect to the haltere disc, as can be shown in Fig. 5. Here, one can see that the orientation of the A and P domains of the haltere disc with respect to the larva is different in early stage larvae as compared to those in the late third instar. This implies a change in the A/P orientation of the haltere disc over time. Since the haltere disc is located beneath the tracheal branch it is attached to, as opposed to the wing disc, which is located above it, tension on the connecting muscle fiber discussed above may be able to induce such a change of orientation in the haltere disc.

Besides the A/P boundary, the wing and haltere discs are separated into dorsal and ventral compartments by a specific D/V boundary [21], [22]. Much less is known about the initial establishment of this D/V boundary [23], [24], [32], [33], thus an in-vivo investigation of the temporal development may shed new light on the formation and control of the D/V boundary in the wing imaginal disc. The dorsal compartment of third instar wing and haltere discs is specified by the expression of the gene apterous, however, at least for the wing disc, the expression of apterous only begins in the middle of the second instar and there are still open questions as to what controls the initial expression of this gene [23], [24]. Both the control of the boundary and the initial expression are connected to the expression of wingless at the end of the wing disc which is furthest from the stalk and will become the ventral end [19], [23], [32], [34]. Moreover, EGFR activation by the gene vein at the opposing end of the tissue close to the stalk has been implicated as a necessary factor for apterous expression [23], [24], [32], [35]. This would imply an initial expression of ap close to the distal end of the disc, where vein signaling is highest. In order to obtain further information on the formation of the D/V boundary, we studied the fluorescent activity of a UAS CD8GFP activated by apterous GAL4, which reflects the promoter activity of this gene. This is shown in Figs. 6 A–K for the left haltere disc and the wing disc (Figs. 7A–J) of the same larvae over time. Images were again taken twice a day with an interval of 8 hours between the images. Due to the variation in growth rates and developmental stage of individual larvae discussed above, a determination of the sequence of expression patterns with time would be difficult to achieve with dissected wing discs.

Interestingly, apterous expression appears to be induced independently in the wing and haltere discs, as well as in the two wing discs of one larva (data not shown). Apterous expression is therefore disc-autonomous. Moreover, the area of initial expression does not coincide with the expression pattern of vein discussed above, but rather lies at the periphery or even outside it. This finding is not concurrent with a direct control of apterous by vein, which in turn is suppressed by wingless [32]. Also, the apterous expression pattern appears to develop from only one or two apterous-expressing cells in both the wing and the haltere disc (see Fig. 8). Again, this does not concur with the permissive region dictated by the combined expression patterns of vein and wingless [21], [32]. Moreover, this indicates that the D/V boundary is not yet established as such when apterous is initially expressed. Rather, it is only established in the early third larval instar. Interestingly, the boundary of the apterous-expressing region is not smooth at this time. The well-defined dorsal-ventral boundary as it is known from late third instar wing and haltere discs does not develop until later in the third instar.

## Discussion and Conclusions

To gain a full picture of imaginal disc growth and patterning it is necessary to be able to observe the development of a single disc over a longer period of time, ideally the full developmental cycle. Here, we have shown that this is possible for the examples of the wing and haltere imaginal discs of Drosophila.

We have seen that growth rates vary over time in individual discs. Using the method for in-vivo imaging introduced above, we have found that the wing imaginal disc is exposed to several sources of tensional forces at different times during development. For example, a large muscle fiber stretches the anterior-ventral compartment around the time of the second molt. At times when growth rates increase these external forces seem to be present. However, further work is needed to elucidate the precise correlation between forces and growth as well as whether the presence of these forces is truly the cause of the increased growth rates. Such a finding would have great implications for the culturing of wing disc tissues in vitro, which does not normally consider such external effects. Also, this scenario would be in accord with recent models of growth control via mechanical feedback, where growth regulation by such forces is stipulated to lead to uniform growth and the determination of final size [6]–[8].

In addition to the growth of the disc, we have also directly studied aspects of pattern formation via the boundaries of the A/P and D/V compartments respectively. The boundary of the A/P compartment is already established in the embryo and remains smooth throughout larval development, as is expected from genetic studies of the regulation of the A/P boundary [31], [36]. The D/V boundary, however, seems to behave very differently. The regulation of this boundary is not known in great detail genetically [19], [20], [35], [37]. However, it has been found that the antagonistic morphogens Wingless and Vein are important regulators of apterous and that in particular the activation of EGFR signaling by Vein seems to be permissive for the expression of apterous [23], [24], [32], [33]. The antagonistic expression patterns of wingless and vein at the end of the disc away from and close to the stalk respectively, where wingless signaling suppresses vein, would predict an initial expression pattern mostly corresponding with that of vein. This is very different from the one we found here using in-vivo imaging, where only one or two cells close to the tracheal branch and far away from the stalk express apterous in the beginning. Moreover, the boundary was generally assumed to be straight from the beginning of apterous expression [34] as is the case for the A/P boundary and engrailed, but this also does not seem to be the case. The D/V boundary is not established immediately following the start of apterous expression. On the other hand, mechanical forces are present at this time in development. In particular, tracheal growth, twist-expressing cells and tiny threads which attach to the posterior half of the wing disc mainly during the second instar may play an important role in the determination of the apterous-expressing region by influencing its growth. However, from our present data we cannot distinguish between the growth of the apterous expressing region by gene induction and that induced by tissue growth. Further work is needed to elucidate this. Also unexpected was the fact that the apterous expression domain does not have a smooth boundary in the beginning of the third larval instar. Further investigations in this direction may shed new light on the control and maintenance of the D/V boundary in the wing imaginal disc.

As we have shown, in-vivo imaging allows for the quantitative study of growth and patterning with fluorescent markers. In the future, this method may be applied to assess both biochemical [5] and biomechanical [6]–[8] models for growth and patterning by feeding such models with data on the temporal development of growth, scaling and temporal changes in morphogen production. This can then lead to a coupled description of the development of growth and form via e.g. mechanical feedback [6]–[8]. Apart from the direct implications on growth control, such a relation between mechanical stresses and growth could also have a profound influence on pattern formation in the disc and its shape. When differently patterned regions have different growth rates, the overall patterning process needs to be described accordingly. Considering the large muscle fiber discussed above, we can speculate that these forces may lead to the change in shape of the *en* expression domain at the boundary between the notum and pouch showing up as a conspicuous kink [38] (see Fig. 4I). This kink also correlates directly with the break in the stripe of dpp expressing cells [39]. Since the muscle fiber exerts a force in the direction away from the posterior compartment before cell proliferation in the wing pouch starts to increase, this mechanical stress may lead to the observed change in the expression pattern if one assumes that stretching leads to cell-proliferation [6]–[8] and that this proliferation is in the direction of the stress [40]. Thus in morphogenesis the influence of growth, patterning and mechanical forces may be very intimately coupled via the time evolution of shape and cell proliferation. An integrated approach, where the temporal evolution of the different factors is taken into account may be very useful in unraveling these interdependencies and give a clearer understanding of morphogenesis, as well as growth.

## Materials and Methods

The following stocks were used for the experiments described:

E-Cad-GFP-III [41]

y w UAS-CD8-GFP;ap-GAL4(CyO); TM6B

y w f; enGAL4/CyO x y [1] w[*]; P{w[+mC] = UAS-mCD8::GFP.L}LL5

Images were processed using ImageJ. Slight larval movement during attainment of the stacks could be corrected for using the stackreg and turboreg plugins [42].

In practice, in a first step, the transmission image of a stack which best showed the wing disc outline was chosen. The fluorescence images were aligned with respect to the corresponding fluorescence image. Slices showing no fluorescent signal were deleted as were slices showing the peripodial membrane if this layer was not of interest. Slices which were taken while the larva moved excessively were also removed and an average z-projection of the fluorescent channel taken. In a final step, this image was superimposed with the chosen transmission image.

To determine the apical disc area, the average z-projection of the aligned fluorescent channel (using ECadGFP-III) was used. Using the ImageJ polygon selections tool the disc area was outlined and the area measured. This area in pixels was then calculated into an area in µm^2^.

Fig. 3B was taken after dissecting the larva in PBS and sticking the wing disc to a glass microscope slide using poly-L-lysine.
